# Utilization of novel systemic therapies for multiple myeloma: A retrospective study of front‐line regimens using the SEER‐Medicare data

**DOI:** 10.1002/cam4.2698

**Published:** 2019-12-04

**Authors:** Daisuke Goto, Rahul Khairnar, Jean A. Yared, Candice Yong, Dorothy Romanus, Eberechukwu Onukwugha, Julia F. Slejko

**Affiliations:** ^1^ Merck Research Laboratories North Wales PA USA; ^2^ Department of Pharmaceutical Health Services Research University of Maryland School of Pharmacy Baltimore MD USA; ^3^ Department of Medicine University of Maryland School of Medicine Baltimore MD USA; ^4^ AstraZeneca Pharmaceuticals LP Gaithersburg MD USA; ^5^ Millennium Pharmaceuticals, Inc., a wholly owned subsidiary of Takeda Pharmaceutical Company Limited Cambridge MA USA

**Keywords:** elderly patients, immunomodulatory drugs (IMiDs), multiple myeloma, proteasome inhibitors, SEER‐Medicare, systemic treatment

## Abstract

The landscape of treatment for multiple myeloma (MM) has significantly changed over the last decade due to novel agents that have shown superiority in efficacy such as proteasome inhibitors (PIs) and immunomodulatory drugs (IMiDs) over traditional therapies. However, the real‐world utilization of these new agents has not been studied well. This study evaluated year‐to‐year changes in treatment choices in a cohort of patients aged 66 or older in the Surveillance, Epidemiology, and End Results (SEER) registry linked with Medicare claims (SEER‐Medicare) data who were diagnosed with MM between 2007 and 2011. We identified 2477 symptomatic newly diagnosed patients who were followed for 6 months or more postdiagnosis and treated with systemic therapies but not with stem cell transplantation. Symptomatic patients were identified by evidence of hypercalcemia, renal failure, anemia, or bone lesions (CRAB criteria). The minimum follow‐up was imposed to ensure sufficient data to characterize treatment. Our analysis found that the proportion of treated patients increased from 75% in the 2007 cohort to 79% in the 2011 cohort. The share of PI‐based regimens including PI plus alkylating agents, PI plus IMiD, and PI‐only increased from 9% to 21%, 3% to 11%, and 16% to 22%, respectively, between 2007 and 2011. These findings translate to the share of PI‐based regimens having increased from 28% to 55% and that of IMiDs‐based regimens (excluding PI plus IMiD) having decreased from 43% to 27%. In conclusion, while the usage of PIs among elderly MM patients increased significantly replacing IMiD‐based regimens (with or without alkylating agents but not with PI) between 2007 and 2011, this significant shift did not increase the proportion of treated patients.

## Graphical abstract

This study evaluated year‐to‐year changes in treatment choices in a cohort of patients aged 66 or older in the Surveillance, Epidemiology, and End Results (SEER)‐Medicare registry diagnosed with MM between 2007 and 2011. We found that while the usage of PIs among elderly MM patients increased significantly replacing IMiD‐based regimens (with or without alkylating agents but not with PI) between 2007 and 2011, this significant shift did not increase the proportion of treated patients.

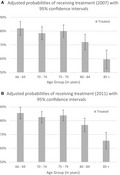

**Key Points**

- A significant increase in usage of novel agents among elderly patients with multiple myeloma (MM) was observed from 2007 through 2011. However, the total proportion of treated patients did not increase, indicating that new therapies replaced old therapies rather than expanding the use of systemic therapies among elderly MM patients.
- The patients' eligibility to receive novel treatments should be evaluated using the latest evidence, and future studies should investigate the best practices for benefiting elderly MM patients with novel agents especially those who have been historically less frequently treated.

## 1 INTRODUCTION

The treatment landscape of multiple myeloma (MM) has changed dramatically over the last two decades. The introduction of immunomodulatory drugs (IMiDs) (such as lenalidomide and thalidomide) and proteasome inhibitors (PIs) (such as bortezomib and carfilzomib) have become increasingly common while the chemotherapy‐based regimens, primarily based on melphalan, have become outdated.^1^, ^2^ In the past, the type of regimen used was based on transplant eligibility, once prohibitive for elderly patients; nowadays, this is a lesser factor. Non‐melphalan‐containing regimens are increasingly used for all MM patients despite the transplant eligibility.^1^ Today, MM risk stratification influences the choice of initial treatment.^3^ Cancer treatment disparities in general and in MM in particular have been documented in the literature. Age and race are among the most studied factors involved in treatment and outcome disparities.^4^, ^5^ Historically, older MM patients were undertreated. Clinical trials have strict inclusion and exclusion criteria and do not give us a clear idea about the real‐world practice patterns and outcomes, while population‐level registry such as SEER‐Medicare can. A retrospective study of elderly diffuse large B‐cell lymphoma (DLBCL) patients from SEER‐Medicare from 2000 to 2007 showed that 23% of the patients did not receive any treatment despite that DLBCL is considered a curable disease.^6^ Now that clinicians have many treatment options for MM, one can tailor the treatment according to the patient's age, comorbidities, safety profile, financial and social burden, and desire to improve outcomes. Therefore, it is important to understand the treatment patterns in elderly patients and find out if elderly MM patients are still undertreated, so we may carefully investigate treatment options available for each patient.

MM is a hematologic malignancy with over 30 000 incident cases in 2016 alone in the United States, ranking the third among hematologic malignancies and 15th among all cancer types.^7^, ^8^ MM is predominantly a disease of the elderly with the median age at diagnosis of 70 years and the 5‐year survival rate over the years between 2006 and 2012 was 50%.^8^, ^9^ MM has been a disease state with significant improvements in treatment options and outcomes in both progression‐free survival and overall survival.^7^, ^10^, ^11^ MM has been traditionally treated with alkylating agents such as melphalan for non‐stem cell transplant patients, of which systemic therapies have been significantly more frequently used among elderly patients.^12^ The majority of changes in treatment of MM, rather, came from the introduction of novel agents including PIs such as bortezomib and IMiDs such as lenalidomide and thalidomide.^13^ Bortezomib and lenalidomide were approved by the FDA for salvage therapies in 2003 and 2005, respectively, and thalidomide was approved for front‐line therapy in 2006.^14^ While the benefits of these therapies on overall and progression‐free survival were unequivocally significant, some adverse effects, such as neuropathy, were found to be more severe than traditional therapies.^15^, ^16^ Although these adverse effects would pose challenges in achieving adherence to treatments, modified regimens have been actively sought for elderly patients since the early stages of clinical adoption.^17^, ^18^, ^19^ Within a few years of introduction, the novel agents have become preferred therapies in the National Comprehensive Cancer Network (NCCN) Guidelines with strong evidence generated through clinical trials, indicating clear superiority in clinical efficacy over traditional alkylating agents while tolerability has been widely observed among elderly patients.^13^, ^17^, ^20^, ^21^, ^22^, ^23^, ^24^

Although patients with MM generally have survival outcomes that exceed many other types of cancer such as lung cancer, a 2010 study indicated significant years of life lost; a published study indicates that patients diagnosed in their 70s lost 11 years and those diagnosed prior to reaching 40 years old lost 36 years.^25^ Clinical trials generated favorable evidence for new therapeutic regimens.^20^, ^21^, ^22^, ^23^ However, fewer studies discussed whether real‐world oncology practices have taken advantage of new therapies. Given the generally poorer survival outcomes among elderly patients, and significant years of life lost, new tolerable treatment options could bring significant treatment benefits to this group. One study found that the use of new agents in initial therapy was closely linked to improved outcomes in elderly patients in a single‐center study.^26^ Yet, there have been major challenges in taking full advantage of new therapies among elderly MM patients due to limited trial results and side effects,^27^ and it is unclear if a significant portion of patients are benefiting from the available new therapies. The aims of the present study were as follows: first, to identify changes in the proportion of patients who received active MM treatment over time and to assess the treatment rate differences between age groups; and second, to evaluate to what extent new therapies have been adopted in real‐world practices.

## 2 METHODS

We analyzed patient‐level clinical and demographic characteristics along with treatment choices recorded in the Surveillance, Epidemiology, and End Results (SEER)‐Medicare database described below.^28^ We compared patients who received systemic therapy with those who did not receive MM‐directed treatment among patients who did not receive a stem cell transplant. This real‐world utilization study addressed whether elderly patients have accessed new therapies and whether new evidence needs to be provided to support better clinical practices.

### 2.1 Data source and inclusion criteria

This was a retrospective study of patients with MM in the SEER‐Medicare database. SEER‐Medicare is a linked dataset that combines the patient‐level clinical data from the SEER registry program and corresponding patients' Medicare claims. This study was reviewed and approved by the University of Maryland Institutional Review Board. We identified patients, who are 66 years or older, who received a diagnosis of MM from 2007 through 2011 in the SEER‐Medicare dataset. This dataset contained billing data for treatments provided to Medicare beneficiaries in the United States linked to cancer registry data from the SEER program by the National Cancer Institute.^28^ The datasets were merged and prepared by the National Cancer Institute. Our dataset included entries dated between 1 January 2006 and 31 December 2012, which is the end of our follow‐up period. We used these data to identify patient‐level clinical and demographic characteristics and clinical events such as administration of systemic therapies. The SEER‐Medicare dataset is regarded as generalizable to the elderly cancer patients.^29^

For this study, we selected patients newly diagnosed with MM (no previous diagnosis), who had continuous enrollment in Medicare Parts A and B for 12 months prior to their MM diagnosis. We also required Part D enrollment for 2 months prediagnosis with a minimum of 6 months of continuous enrollment in the Medicare Parts A, B, and D postdiagnosis. This criterion was designed for complete characterization of first‐line therapies. Patients were followed until death, or until being censored due to loss of Medicare Parts A or B coverage, or end of data availability. We sought to identify patients with symptomatic MM. Since SEER does not differentiate smoldering and symptomatic MM, we only included patients who had evidence of hypercalcemia, renal insufficiency, anemia, and bone (CRAB) symptoms and any associated therapies based on claims data found in the 6‐month period preceding the diagnosis and within the month of, and month following, diagnosis. The CRAB symptoms relied on National Drug Code (NDC), ICD‐9, and the Healthcare Common Procedure Coding System (HCPCS) codes reported in medical billing records associated with CRAB symptoms. For the purpose of identifying diseases in bone lesions and associated therapies, we identified bone fractures, bone diseases, use of radiation, and denosumab.

### 2.2 Identification of treatments

Using medical and pharmacy claims data, NDC and HCPCS codes, we identified the following as MM‐directed therapy: PIs (bortezomib), IMiDs (lenalidomide and thalidomide), and alkylating agents (melphalan, cyclophosphamide, vincristine, and bendamustine). Corticosteroids (dexamethasone and prednisone) could have been used along with any of the aforementioned therapies. We excluded patients who initiated MM‐directed therapies prior to the month of diagnosis.

Treatment lines were determined using a previously developed treatment algorithm described below (with details in Appendix A). The algorithm was developed in collaboration with several hematology/oncology specialists to proxy the definition of a line of treatment within the randomized controlled trials and in accordance with the NCCN Guidelines for treatment of MM.^30^, ^31^ This algorithm has been used for several recent observational studies of MM treatment.^32^, ^33^, ^34^, ^35^, ^36^ The first date of MM‐directed treatment (TX) was the date in which first‐line therapy was initiated. Administration or dispensing of MM‐directed treatment was considered continuous as long as the same set of drugs was repeated without more than 60 days of discontinuation. Starting at the initiation of therapy, we created treatment episodes (TXEs) constituted of all agents received within 30 days following the first fill date or first day of infusion for an MM‐directed agent. An addition of a new agent to this combination or a treatment gap of >60 days after the run out date (30 days after the last day of supply or last day of infusion) of the last agent in the TXE marked the beginning of a new TXE. Administration of corticosteroids was not considered to be part of MM‐directed treatments if administered in a combination with any other agents. However, single agent dexamethasone, if given for >90 days, constituted a TXE. Other use of steroids alone was classified as a steroid burst and not included in TX. First‐line treatment was identified from the TXEs: A gap of >90 days between the run out date of a TXE (TXE n) and a subsequent TXE (TXE n + 1) marked the beginning of a new line of treatment while the run out date for the TXE n marked the end of the first‐line treatment. The first‐line treatment was classified into six therapeutic regimen groups: PI‐IMiDs (consists of one PI and one IMiDs), PI‐alkylating agent (one PI and one alkylating agents), IMiDs (one IMiD), PI (one PI), IMiDs‐alkylating agent (one IMiD and one alkylating agent), and any other drug combinations (single agent as well as combination therapies).

### 2.3 Study variables

Baseline characteristics at the time of diagnosis included the date of diagnosis (identified by SEER), SEER registry region (West, Northeast, South, Mid‐West), age at diagnosis, race/ethnicity (Caucasian (non‐Hispanic), African‐American (non‐Hispanic), and other), and marital status (currently married or not married/unknown). We used the Charlson Comorbidity Index (CCI) to identify patients' comorbidities and their severity using the claims data 1 year preceding diagnosis.^36^ We used a published algorithm to identify indicators for poor performance using the claims data; the indicators used in identification included health, hospital, hospice, skilled nursing facilities, oxygen, walking aids, and wheelchairs within one preceding year of diagnosis.^37^ Dual eligibility indicates the patient's eligibility to receive the Medicaid coverage anytime in the calendar year preceding to the year of diagnosis. Clinical events, such as a drug administration, were identified from the Medicare claims; and vital statistics, such as death, were identified primarily from the SEER portion of the dataset in conjunction with the Medicare portion for missing data.

### 2.4 Statistical analysis

We used descriptive statistics to characterize the sample population and adjusted statistical analysis to estimate the overall probability of receiving at least one line of systemic treatment and probabilities of receiving regimens aforementioned in Section 2.2 as a front‐line treatment. Chi‐square statistics were used in unadjusted analysis to estimate the statistical significance of differences in the categorical baseline characteristics described in Section 2.3. The probability of receiving any MM‐directed initial treatment following a diagnosis was computed using a logistic model adjusted for the aforementioned baseline characteristics. A subsequent analysis investigated the probability of receiving any of the six therapeutic regimens discussed in Section 2.2 using the multinomial logistic model adjusted for baseline characteristics. A Hausman‐type simultaneous equation test was conducted to test whether the availability of alternative treatment options does not cause statistical biases in analysis to ensure the validity of our results.^38^ Our results are reported as the excess (marginal) probability of receiving a therapy compared with the reference group population and differences in probabilities (percentage [%] points) between the reference groups and other groups. We computed average marginal effects. Therefore, the changes are average changes experienced by our sample. Reference groups for diagnosis year was 2007, 66‐70 for age, non‐Hispanic for race, male for sex, not married for marital status, West for region, no for Medicare and Medicaid dual eligibility, no for poor performance indicators, and 0 for CCI. We conducted the following sensitivity analysis to ensure the validity of our statistical mode. The first additional model included interaction terms between age and numeric diagnosis year, and the second additional model included interaction terms between age and diagnosis year (categorical). We used STATA 12 (StataCorp, LLC, College Station, Texas) for statistical analysis.

## 3 RESULTS

**Figure 1:**
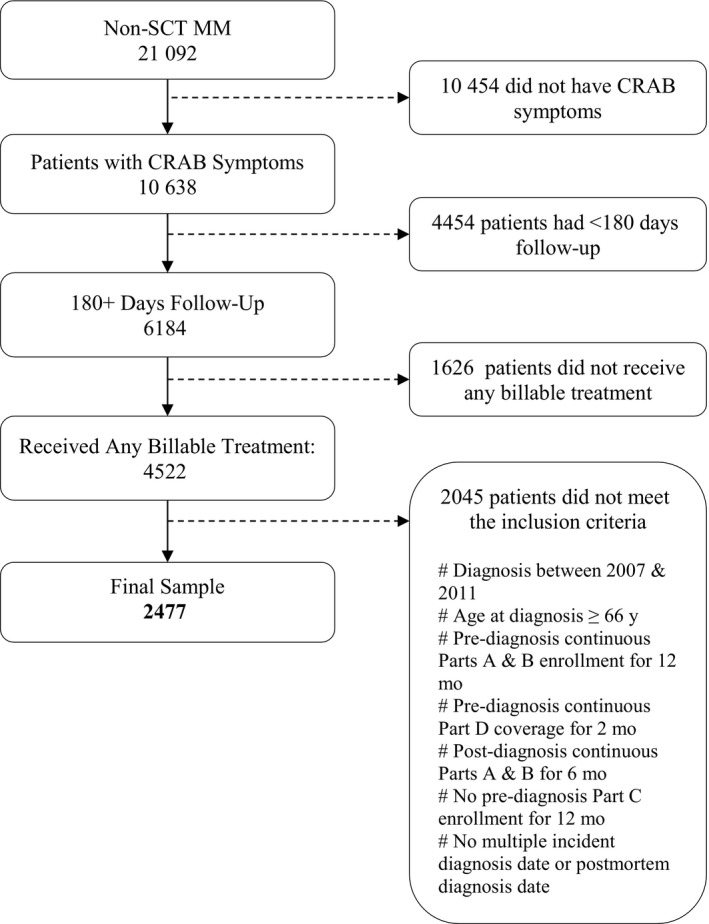
Sample selection

After applying the eligibility criteria, our sample consisted of 2477 MM patients (Figure 1). Among them, 1935 patients (78%) received systemic therapy and 542 patients (22%) did not receive MM‐directed therapy (Table 1). Among 1935 treated patients, IMiD therapy predominated (563 [29%] patients), followed by PI therapy (397 [20%]), PI plus IMiD combinations (271 [14%]), alkylating agents (171 [9%]), PI and an alkylating agent‐based regimen (160 patients [8%]), and an IMiD plus alkylating agent combinations (119 [6%]) (Table 2). The majority of the remaining 254 patients (13%) received either a combination treatment with bortezomib and doxorubicin, dexamethasone monotherapy, or combinations with three or more MM‐directed therapies (69 [27%], 84 [33%], and 61 [24%] patients of the 254 remaining patients, respectively).

**Table 1:** Baseline characteristics of MM patients by treatment status

TX status	NOTX		TX		Total		*P*‐Value
	N	Row %	N	Row %	N	Row %	
	542	22	1935	78	2477	100	
	N	Col%	N	Col%	N	Col%	
Age							
66‐69	57	15	317	85	374	15	**<.01**
70‐74	112	18	511	82	623	25	
75‐79	104	17	495	83	599	24	
80‐84	120	25	365	75	485	20	
85+	149	38	247	62	396	16	
Sex							
Male	252	46	932	48	1184	49	.49
Female	290	54	1003	52	1293	51	
Race							
Non‐Hispanic White	398	73	1528	79	1926	78	**<.01**
Other	144	27	407	21	551	22	
Marital status							
No indication	338	62	941	49	1279	50	**<.01**
Married	204	38	994	51	1198	50	
Region							
W (West)	206	38	849	44	1055	42	**<.01**
NE (Northeast)	133	25	333	17	466	19	
MW (Midwest)	62	11	286	15	348	14	
S (South)	141	26	467	24	608	25	
Diagnosis year							
2007	111	20	337	17	448	18	.59
2008	96	18	359	19	455	18	
2009	106	20	381	20	487	20	
2010	118	22	444	23	562	23	
2011	111	20	414	21	525	21	
Poor performance indicator							
No	264	49	1141	59	1405	58	**<.01**
Yes (Confirmed)	278	51	794	41	1072	42	
Medicare and medicaid dual eligibility							
Not dual eligibility	348	64	1395	72	1743	72	**<.01**
Dual eligibility	194	36	540	28	734	28	
CCI 12 months prior diagnosis							
0 or miss	150	28	798	41	948	40	**<.01**
1	107	20	441	23	548	22	
2+	285	52	696	36	981	38	

Bold faced numbers are statistically significant at the 95% level.

N = number of patient.

**Table 2:** Baseline characteristics of MM patients by treatment regimens

Regimen	PI + ALK		PI + IMiDs		PI		IMiDs		IMiDs + ALK		Other		Total		*P‐*value
	N	Row %	N	Row %	N	Row %	N	Row %	N	Row %	N	Row %	N	Row %	
	271	14	160	8	397	21	563	29	119	6	425	22	1935	100	
	N	Col %	N	Col %	N	Col %	N	Col %	N	Col %	N	Col %	N	Col %	
Age															
66‐69	69	25	22	14	61	15	82	15	14	12	69	16	317	16	**<.01**
70‐74	81	30	40	25	102	26	159	28	33	28	96	23	511	26	
75‐79	60	22	57	36	100	25	132	23	26	22	120	28	495	26	
80‐84	38	14	24	15	84	21	114	20	31	26	74	17	365	19	
85+	23	8	17	11	50	13	76	14	15	13	66	16	247	13	
Sex															
Male	140	52	80	50	206	52	255	45	46	39	205	48	932	48	**.05**
Female	131	48	80	50	191	48	308	55	73	61	220	52	1003	52	
Race															
Non‐hispanic white	225	83	124	78	316	80	449	80	80	67	334	79	1528	79	**.02**
Other	46	17	36	23	81	20	114	20	39	33	91	21	407	21	
Marital status															
No indication	115	42	75	47	191	48	288	51	61	51	211	50	941	49	.27
Married	156	58	85	53	206	52	275	49	58	49	214	50	994	51	
Region															
W (West)	128	47	70	44	171	43	242	43	50	42	188	44	849	44	**<.01**
NE (Northeast)	55	20	18	11	75	19	109	19	14	12	62	15	333	17	
MW (Midwest)	23	9	40	25	65	16	64	11	19	16	75	18	286	15	
S (South)	65	24	32	20	86	22	148	26	36	30	100	24	467	24	
Diagnosis year															
2007	31	9	11	3	54	16	114	34	30	9	97	29	337	17	**<.01**
2008	43	12	24	7	65	18	104	29	33	9	90	25	359	19	
2009	40	10	34	9	90	24	112	29	17	4	88	23	381	20	
2010	69	16	47	11	95	21	133	30	26	6	74	17	444	23	
2011	88	21	44	11	93	22	100	24	13	3	76	18	414	21	
Poor performance indicator															
No	175	65	91	57	239	60	337	60	58	49	241	57	1141	59	**.01**
Yes (Confirmed)	96	35	69	43	158	40	226	40	61	51	184	43	794	41	
Medicare and medicaid dual eligibility															
Not dual eligibility	205	76	120	75	305	77	404	72	66	56	295	69	1395	72	**<.01**
Dual eligibility	66	24	40	25	92	23	159	28	53	45	130	31	540	28	
CCI 12 months prior diagnosis															
0 or miss	120	44	66	41	165	42	227	40	51	43	169	40	798	41	.24
1	70	26	31	19	76	19	127	23	32	27	105	25	441	23	
2+	81	30	63	39	156	39	209	37	36	30	151	36	696	36	

Bold faced numbers are statistically significant at the 95% level.

N = number of patient.

In univariate analyses (Table 1), we found an association between age and likelihood of treatment receipt. The proportion of patients who were treated decreased from 85% to 62% for age groups 66‐69 and 85 + years old, respectively (*P* < .01). Year of diagnosis and gender were not significant predictors (*P* = .59 and .49) of treatment. The likelihood of receiving treatment was statistically higher for married persons, non‐dual eligible patients, White patients, and those with a lower comorbidity burden (CCI, all *P* < .01). Patients with a poor performance indicator and those residing in Northeast had a lower probability of receiving treatment (*P* < .01, Table 1).

Among treated patients, the share of all regimens that included a PI increased over the years 2007 through 2011 (9% to 21%, 3% to 11%, and 16% to 22% for PI plus alkylating agents, PI plus an IMiD, and PI‐only, respectively). While the use of IMiD in combination with a PI increased by 8% points between 2007 and 2011, IMiD‐based regimens not including a PI declined over the same period (34% to 24% and 9% to 3% for IMiD‐only and IMiD and alkylating agents, respectively) (Table 2). This finding also indicates that the use of alkylating agents has declined over time.

Aggregated over the 2007 cohort through 2011, PI plus an alkylating agent was less likely to be administered in older patients (22% of all regimens in the 66‐69 age group vs 9% among 85 + years old patients), whereas PI‐only and IMiD‐only regimen distribution did not vary significantly by age (Table 2). In multivariate analysis, we found that age was significantly associated with the chance of receiving treatment (80‐85 age group: 9% points [*P* = .001] lower; 86 + age group: 20.8% points [*P*‐value < .001] lower than the 66‐70 age group) after controlling for other confounding factors. Year of diagnosis was not statistically associated with treatment receipt. The likelihood of married patients to receive a treatment was 6.4% points higher (*P* < .001) than their unmarried counterparts, and a higher comorbidity burden (CCI score of 2+) was associated with a 10.2% points lower (*P* < .001) likelihood of receiving treatment. Regional differences in treatment patterns were also noted, with the Northeast region associated with a lower propensity for treatment (7.7% points lower than the West region [*P* = .001]). There was a trend of a lower likelihood of treatment among non‐Hispanic Black patients compared to White patients (4.3% points lower [*P* = .077]), but the difference did not meet statistical significance. Other baseline characteristics, including gender, dual eligibility with Medicaid, and poor performance status, were not associated with treatment receipt (Table 3). These results are based on the base model. There were no scientifically or clinically important differences between the base analysis and sensitivity analysis. Since the base model is more parsimonious than the alternative specifications, we continue to discuss our results using the base model.

**Table 3:** Multinomial logit model for treatment choice

Total N analyzed = 2477	Treatment choice (Treatment vs No Treatment) Average Marginal Effects (differences in probability) (% points)	
	Marginal Chance of Being Treated	*P* value
Diagnosis year (Reference category = 2007)		
2008	3.2%	.242
2009	2.5%	.352
2010	3.9%	.129
2011	4.1%	.118
Age (Reference category = 66‐70)		
71‐75	−3.1%	.196
76‐80	−1.8%	.454
81‐85	**−9.0%**	**.001**
86 +	**−20.8%**	**<.001**
Race (Reference category = Non‐hispanic white)		
Non‐hispanic black	−4.3%	.077
Other	0.8%	.822
Sex (Reference category = Male)		
Female	2.5%	.151
Marital status (Reference category = Not Married)		
Married	**6.4%**	**<.001**
Region (Reference category = West)		
Northeast	**−7.7%**	**.001**
Midwest	1.3%	.597
South	−2.9%	.170
Medicare and medicaid dual eligibility (Reference Category = No)		
Yes	−2.8%	.156
Poor performance indicator (Reference category = No)		
Yes	−1.9%	.276
CCI 12 months prior diagnosis (Reference category = 0)		
1	−2.2%	.285
2+	**−10.2%**	**<.001**

Bold faced numbers are statistically significant at the 95% level.

**Figure 2:**
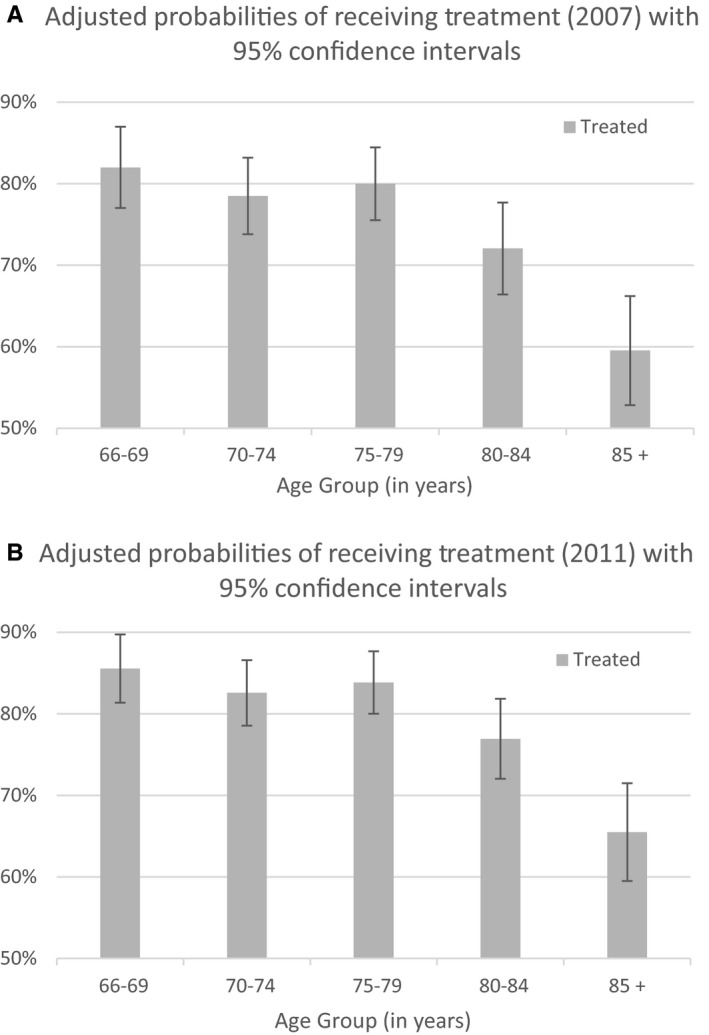
Adjusted probabilities of receiving treatment. A, Adjusted probabilities of receiving treatment (2007) with 95% confidence intervals. B, Adjusted probabilities of receiving treatment (2011) with 95% confidence intervals

The adjusted estimates of probabilities of receiving a treatment for patients diagnosed in 2007 and 2011 (Figure 2; Appendix Figure A for 2008‐2010, and Appendix Table A for estimated probabilities) show minimal differences. For patients diagnosed in 2007, some 82% (the 95% confidence interval [CI]: [77%, 87%]) of the age group 66‐69 and 60% (CI:[53%, 66%]) of the age group 85 + received a treatment. For the 2011 cohort, the adjusted probabilities were 86% (CI:[81%, 90%]) and 65% (CI:[59%, 71%]). We note that these confidence intervals significantly overlaps indicating statistical insignificance of the increase.

In the analysis of treatment choices, a significant time trend between the year of diagnosis and increased use of PI‐based treatments was noted. The probability of receiving PI‐alkylating, PI‐IMiD, and PI‐only regimens increased by 12.7% points (*P* < .001), 7.3% points (*P* < .001), and 6.4% points (*P* = .025) between the 2007 and 2011 cohorts according to the estimates based on the base model (Table 4). Our results satisfied independence of irrelevant alternatives; Hausman tests were conducted to compare the full sample and subsamples from which one treatment group was removed at a time^27^; for all subsamples, Hausman test *P*‐values were > .05. Older age was associated with a decreased use of PI plus alkylating agents only with a 13.2% point decrease for 86 + year old compared to the 66‐70 years old group (*P* < .001). Dual eligibility and poor performance status seemed to influence treatment choice with IMiD and alkylating agent combinations but not with other therapy types. Specifically, dual eligible patients and those with poor performance status had a 4.3% (*P* = .006) and 2.3% (*P* = .061) point higher probability of treatment receipt with an IMiD/alkylating agent combination compared to their counterparts (ie, non‐dual eligible patients and those without poor performance status, respectively). Interestingly, comorbidity burden as measured by the CCI did not appear to influence treatment type. For other covariates, statistical significance was not generally observed. The results discussed above were consistent with the findings from the alternative models.

**Table 4:** Multinomial logit model for regimen choice

Total N analyzed = 1935	PI + ALK		PI + IMiDs		PI		IMiDs^a^		IMiDs + ALK	
	Marginal chance of receiving this treatment (% points)	*P* value	Marginal chance of receiving this treatment (% points)	*P* value	Marginal chance of receiving this treatment (% points)	*P* value	Marginal chance of receiving this treatment (% points)	*P* value	Marginal chance of receiving this treatment (% points)	*P* value
Diagnosis Year (Reference category = 2007)										
2008	2.7%	.228	**3.6%**	**.030**	2.4%	.403	−4.5%	.195	−0.4%	.836
2009	1.7%	.429	**5.4%**	**.002**	**7.4%**	**.011**	−3.9%	.258	**−4.4%**	**.020**
2010	**6.9%**	**.003**	**7.4%**	**<.001**	**5.5%**	**.049**	−3.8%	.256	−3.3%	.085
2011	**12.7%**	**<.001**	**7.3%**	**<.001**	**6.4%**	**.025**	**−9.5%**	**.004**	**−5.7%**	**.001**
Age (Reference category = 66‐70)										
71‐75	**−6.4%**	**.022**	1.0%	.598	0.7%	.815	5.3%	.098	2.0%	.178
76 −80	**−9.7%**	**<.001**	**4.6%**	**.022**	0.7%	.804	0.5%	.878	1.0%	.506
81 −85	**−11.6%**	**<.001**	−0.5%	.780	3.2%	.309	5.1%	.147	**4.5%**	**.014**
86 +	**−13.2%**	**<.001**	−0.1%	.971	0.4%	.905	3.9%	.311	2.2%	.257
Race (Reference category = Non‐Hispanic White)										
Non‐Hispanic Black	**−4.7%**	**.030**	3.3%	.155	5.2%	.101	−4.0%	.189	−0.2%	.902
Other	0.1%	.984	0.2%	.943	−4.2%	.249	−0.5%	.913	**7.2%**	**.024**
Sex (Reference category = Male)										
Female	−0.9%	.581	−0.5%	.709	−3.1%	.116	3.1%	.154	1.7%	.128
Marital Status (Ref = Not Married)										
Married	1.7%	.305	0.1%	.944	−0.8%	.679	−1.8%	.426	0.9%	.470
Region (Reference category = West)										
Northeast	2.8%	.244	−3.1%	.053	0.8%	.781	3.4%	.261	−0.4%	.779
Midwest	**−6.5%**	**.001**	**4.7%**	**.033**	0.6%	.844	**−6.3%**	.035	2.6%	.169
South	0. 5%	.818	−1.9%	.213	−3.0%	.206	3.4%	.220	2.7%	.087
Medicare and medicaid dual eligibility (Reference category = No)										
Yes	−1.2%	.522	−1.8%	.229	**−5.0%**	.023	0.6%	.811	**4.3%**	**.006**
Poor performance indicator (Reference category = No)										
Yes	−1.7%	.327	0.7%	.629	−1.1%	.600	−2.0%	.386	2.3%	.061
CCI 12 months prior diagnosis (Reference category = 0)										
1	1.0%	.637	−1.0%	.519	−3.0%	.188	0.2%	.952	−0.1%	.927
2+	−2.6%	.174	0.3%	.858	2.3%	.316	2.2%	.382	−2.5%	.056

Bold faced numbers are statistically significant at the 95% level.

a Reference category for multinomial logit estimation.

**Figure 3:**
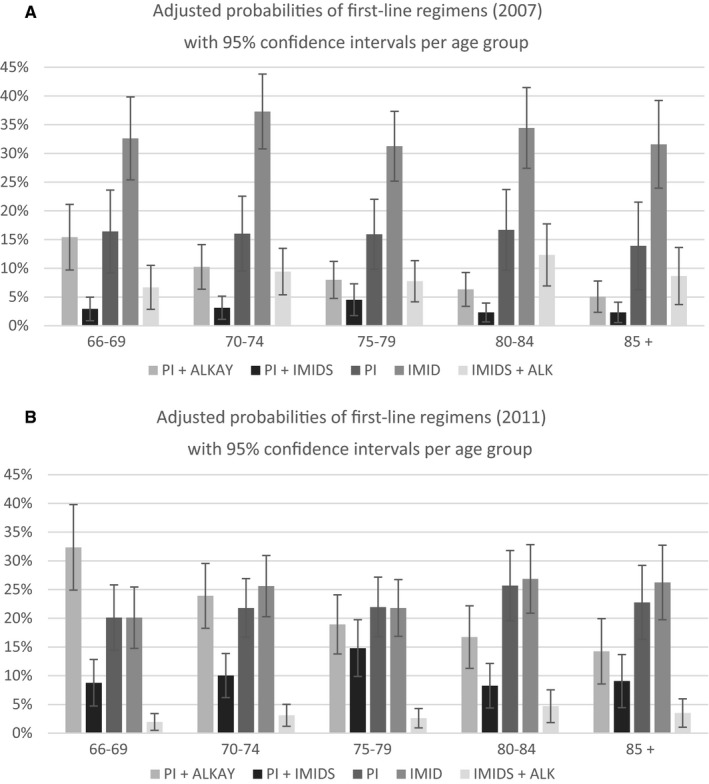
Adjusted probabilities of first‐line regimens. A, Adjusted probabilities of first‐line regimens (2007) with 95% confidence intervals per age group. B, Adjusted probabilities of first‐line regimens (2011) with 95% confidence intervals per age group

The adjusted estimates of treatment regimens for patients diagnosed in 2007 and 2011 (Figure 3; Appendix B for 2008‐2010) show large differences consistent with the findings above. In 2007, some 15% (CI: [10%,21%]) of the lowest age group 66‐69 and 5% (CI:[2%,8%]) of the highest age group 85+ received PI‐alkylating after adjusting for other factors. For the 2011 cohort, the adjusted probabilities were 32% (CI:[25%, 40%]) and 14% (CI:[9%, 20%]). The adjusted probabilities of receiving PI‐IMiD regimen were 3% (CI:[1%, 5%]) and 2% (CI:[1%, 4%]) for the lowest and highest age groups among those diagnosed in 2007, and 9% (CI:[5%, 13%]) and 9% (CI:[5%, 14%]), respectively, for the 2011 cohort. The adjusted probabilities of receiving PI regimen were 16% (CI:[11%, 22%]) and 14% (CI:[9%, 19%]) for the lowest and highest age groups in 2007, and 20% (CI:[14%, 26%]) and 23% (CI:[16%, 29%]), respectively, for the 2011 cohort.

### 3.1 Discussion

Among 2477 patients in our sample, 1935 patients (78%) received systemic therapy and 542 patients (22%) did not receive any MM therapy within 6 months from diagnosis. Over the course of 5 years during the study period, no statistically significant change was observed in the proportion of treated patients. The adjusted probabilities of being treated for patients diagnosed in 2007 and 2011 (Figure 2 and Appendix Table A for numeric results) also show small changes (4% or 5% points) across all age groups confirming the finding. Older patients' survival is known to be inferior to younger patients^26^; this fact might be reflected in the share of untreated patients. Our multivariate analysis found factors that indicate a lesser chance of being treated for age over 80 years old (−9.0% points [*P* = .001]; for age 86+, −20.8% points [*P* < .001]), not being married (−6.4% points [*P* < .001]), living in the Northeast region (−7.7% points [*P* = .001]), and with CCI greater or equal to two (−10% points[*P* < .001]) (Table 3).

Among those who received a treatment, treatments that are better tolerated than multidrug regimens such as monotherapies with an IMiD or PI were the most commonly administered regimens (accounting for 50% of all regimens administered overall; see Table 2), while more effective treatment combinations, such as PI‐based regimens combined with alkylating agents or IMiDs, were less frequently observed in this older population (22% of all administrations) (Table 2). The multivariate analysis indicated that PI plus alkylating agent regimen was incrementally less utilized for patients in higher age categories (−6.4% points for age 71‐75 [*P* = .022], and −13.2% for age 86 or older [*P* < .001]) compared with age group 66‐69 while age was not a factor for receiving other regimens (see Table 4). This finding also indicates that elderly patients tend to avoid multidrug therapies involving PI.

Over the period between 2007 and 2011, frequently used regimens quite significantly changed. It was found that PI‐based regimens were found to be used more frequently over the cohort years 2007 and 2011 (increased from 25% of all regimens to 54% over these years), and IMiDs‐based regimens that do not include PIs became less popular over the same period (43% to 27%) (Table 2). Our multivariate analysis also found consistent results (Table 4). Compared with the 2007 cohort, patients diagnosed in 2011 were 12.7% points more likely [*P* < .001] to receive the PI plus alkylating drug combinations after controlling for other confounding factors, 7.3% points more likely [*P* < .001] to receive PI plus IMiD, 6.4% points more likely [*P* = .025] to receive PI‐only, but 9.5% points less likely [*P* = .004] to receive IMiD‐only, and 5.7% points less likely [*P* = .001] to receive IMiD plus alkylating agents. The adjusted probabilities also indicate that the share of PI‐based regimens sharply increased and the share of IMiD therapies sharply decreased over time across all age groups (See Figure 3 and Appendix Table B for numeric results). These findings are consistent with the accumulation of evidence over diagnosis years 2007 through 2011 for the survival benefits of PI‐based regimens. Our results also indicate that IMiDs are increasingly used in a combination regimen with a PI. A previous study found a declining trend of treatment with IMiD‐only over time among patients who were diagnosed in 2007, 2008, and 2009.^39^ The same study reported that IMiDs maintained a relatively stable share over the three cohorts (0.8% point decline) and we also found a relatively stable share over the same three cohorts (4.0% points).

While it is clear that novel PI‐based regimens replaced IMiD‐based therapies over time, it is notable that our results indicate that the proportion of treated patients did not increase during the same period (Table 3; no coefficients for diagnosis year were statistically significant), indicating that novel agents did not extend therapeutic options to a larger patient population.

While it is true that with novel agents patients are more likely to experience adverse events, there have been numerous studies and trials that demonstrated ways to maximize the benefits of PI‐based and novel regimens.^40^, ^41^, ^42^, ^43^ The benefits of novel therapies have been actively studied; the choice of treatment regimens has been based on age, as our results confirm, risk stratification, performance status, and comorbidities.^44^, ^45^ Although there has been more than a decade of continued discussions on ways to mitigate the risk of adverse events and side effects of novel agents,^40^ recent studies still identified that further investigations are needed to develop best practices for customizing the dosing schemes to mitigate toxicity risk among elderly and frail patients with concomitant comorbidities.^44^, ^45^, ^46^, ^47^ Challenges remain in individualizing treatment choices that minimize the risk to benefit ratio. Our study found that the introduction of new therapies did not increase the share of treated patients in the geriatric population; and this finding is consistent with continued challenges in utilizing novel agents in frail and elderly patients. Our analysis supports continued efforts in developing best practices for active treatments in the geriatric population for whom treatments have historically been less likely to be extended in usual clinical settings.

### 3.2 Limitations

Our study has several limitations. First of all, our study was not designed to characterize patients' entire treatment pathway. Our study cohort received diagnosis up to and including 2011 and we had records for only 1 to 2 years beyond this. 85% and 55% of patients were followed until death for those diagnosed in 2007 and 2011, respectively. Among those who received a systemic therapy as a front‐line therapy, 82% and 52% were followed until death, respectively. The purpose of our study was to characterize treatments received by patients and it was possible that some of the patients could have been still in treatment at the time of their last observations in our data. Further studies are needed to identify the impact of treatment on outcomes such as overall and progression‐free survival. In the treatment of MM, risk factors, such as cytogenetic abnormalities, are used to make treatment decisions; however, due to the lack of this information in this dataset, our study did not examine this. Therefore, our results should not be seen as translatable to subpopulation‐based risk profiles. Additionally, we identified 919 patients who had less than 6 months of follow‐up before death or a loss of Medicare A and B benefits; among them, only 33% of patients were found to have had any treatment. Our objective of this study was to identify treatment among those who had a minimum of a 6‐month follow‐up to observe the intended full course of front‐line treatment; the survivorship bias to the probability of receiving treatment is not resolved in this study.

## 4 CONCLUSION

New treatment regimens quickly changed the treatment landscape for MM patients over the last decade. This study revealed that traditional therapies were replaced with newer agents among patients who underwent treatment over the years 2007 through 2011; however, availability of new agents with a more favorable toxicity profile did not increase the proportion of treated patients. Our results call for continued investigation on ways to expand the utilization of novel therapies in real‐world settings among elderly patients.

## Data Availability

The data that support the findings of this study are available from the National Cancer Institute. Restrictions apply to the availability of these data, which were used under license for this study.
