# Supplementary material for: Utilization of novel systemic therapies for multiple myeloma: A retrospective study of front‐line regimens using the SEER‐Medicare data
Source: Cancer Med. 2019 Dec 4;9(2):626–39. doi: 10.1002/cam4.2698 (PMC6970041; doi:10.1002/cam4.2698)
Supplement: Supplementary file 1 [file CAM4-9-626-s001.pdf]

Appendix Table A. Probability of receiving a treatment (Adjusted) by age group

YEAR OF DIAGNOSIS == 2007

	Probability	CI 95% LOW	CI 95% HIGH
66 - 69	82%	77%	87%
70 - 74	78%	74%	83%
75 - 79	80%	76%	84%
80 - 84	72%	66%	78%
85 +	60%	53%	66%

YEAR OF DIAGNOSIS == 2008

	Probability	CI 95% LOW	CI 95% HIGH
66 - 69	85%	81%	90%
70 - 74	82%	78%	86%
75 - 79	83%	79%	87%
80 - 84	76%	71%	81%
85 +	65%	58%	71%

YEAR OF DIAGNOSIS == 2009

	Probability	CI 95% LOW	CI 95% HIGH
66 - 69	85%	80%	89%
70 - 74	81%	77%	86%
75 - 79	83%	79%	87%
80 - 84	76%	70%	81%
85 +	64%	57%	70%

YEAR OF DIAGNOSIS == 2010

	Probability	CI 95% LOW	CI 95% HIGH
66 - 69	85%	81%	89%
70 - 74	82%	78%	86%
75 - 79	83%	80%	87%
80 - 84	76%	71%	81%
85 +	65%	59%	71%

YEAR OF DIAGNOSIS == 2011

	Probability	CI 95% LOW	CI 95% HIGH
66 - 69	86%	81%	90%
70 - 74	83%	79%	87%
75 - 79	84%	80%	88%
80 - 84	77%	72%	82%
85 +	65%	59%	71%

Appendix Table B. Treatment Choice (Adjusted) by age group

YEAR OF DIAGNOSIS = 2007
REGIMEN = PI_ALKAY

	Probability	CI 95% LOW	CI 95% HIGH
66 - 69	15%	10%	21%
70 - 74	10%	6%	14%
75 - 79	8%	5%	11%
80 - 84	6%	3%	9%
85 +	5%	2%	8%

YEAR OF DIAGNOSIS = 2007
REGIMEN = PI_IMIDS

	Probability	CI 95% LOW	CI 95% HIGH
66 - 69	3%	1%	5%
70 - 74	3%	1%	5%
75 - 79	5%	2%	7%
80 - 84	2%	1%	4%
85 +	2%	1%	4%

YEAR OF DIAGNOSIS = 2007
REGIMEN = PI

	Probability	CI 95% LOW	CI 95% HIGH
66 - 69	16%	11%	22%
70 - 74	16%	11%	21%
75 - 79	16%	11%	21%
80 - 84	17%	12%	22%
85 +	14%	9%	19%

YEAR OF DIAGNOSIS = 2007
REGIMEN = IMID

	Probability	CI 95% LOW	CI 95% HIGH
66 - 69	33%	25%	40%
70 - 74	37%	31%	44%
75 - 79	31%	25%	37%
80 - 84	34%	27%	41%
85 +	32%	24%	39%

YEAR OF DIAGNOSIS = 2007
REGIMEN = IMIDS_ALKAY

	Probability	CI 95% LOW	CI 95% HIGH
66 - 69	7%	3%	11%
70 - 74	9%	5%	13%
75 - 79	8%	4%	11%
80 - 84	12%	7%	18%
85 +	9%	4%	14%

Appendix Table B. Treatment Choice (Adjusted) by age group

YEAR OF DIAGNOSIS = 2008
REGIMEN = PI_ALKAY

	Probability	CI 95% LOW	CI 95% HIGH
66 - 69	19%	13%	25%
70 - 74	13%	9%	17%
75 - 79	10%	7%	14%
80 - 84	8%	5%	12%
85 +	7%	3%	10%

YEAR OF DIAGNOSIS = 2008
REGIMEN = PI_IMIDS

	Probability	CI 95% LOW	CI 95% HIGH
66 - 69	6%	3%	9%
70 - 74	7%	4%	10%
75 - 79	10%	5%	14%
80 - 84	5%	2%	8%
85 +	5%	2%	8%

YEAR OF DIAGNOSIS = 2008
REGIMEN = PI

	Probability	CI 95% LOW	CI 95% HIGH
66 - 69	18%	12%	23%
70 - 74	18%	13%	23%
75 - 79	18%	13%	23%
80 - 84	20%	14%	25%
85 +	17%	11%	23%

YEAR OF DIAGNOSIS = 2008
REGIMEN = IMID

	Probability	CI 95% LOW	CI 95% HIGH
66 - 69	27%	20%	33%
70 - 74	32%	26%	37%
75 - 79	27%	21%	32%
80 - 84	30%	24%	37%
85 +	29%	22%	36%

YEAR OF DIAGNOSIS = 2008
REGIMEN = IMIDS_ALKAY

	Probability	CI 95% LOW	CI 95% HIGH
66 - 69	6%	3%	10%
70 - 74	9%	6%	13%
75 - 79	8%	4%	11%
80 - 84	13%	8%	18%
85 +	9%	4%	15%

Appendix Table B. Treatment Choice (Adjusted) by age group

YEAR OF DIAGNOSIS = 2009
REGIMEN = PI_ALKAY

	Probability	CI 95% LOW	CI 95% HIGH
66 - 69	17%	11%	23%
70 - 74	12%	8%	16%
75 - 79	9%	6%	13%
80 - 84	8%	5%	11%
85 +	7%	3%	10%

YEAR OF DIAGNOSIS = 2009
REGIMEN = PI_IMIDS

	Probability	CI 95% LOW	CI 95% HIGH
66 - 69	8%	4%	11%
70 - 74	8%	5%	12%
75 - 79	12%	8%	17%
80 - 84	7%	4%	10%
85 +	7%	3%	11%

YEAR OF DIAGNOSIS = 2009
REGIMEN = PI

	Probability	CI 95% LOW	CI 95% HIGH
66 - 69	22%	16%	29%
70 - 74	23%	18%	29%
75 - 79	23%	18%	28%
80 - 84	26%	20%	32%
85 +	23%	16%	30%

YEAR OF DIAGNOSIS = 2009
REGIMEN = IMID

	Probability	CI 95% LOW	CI 95% HIGH
66 - 69	26%	20%	33%
70 - 74	32%	26%	38%
75 - 79	27%	21%	32%
80 - 84	32%	25%	38%
85 +	31%	24%	38%

YEAR OF DIAGNOSIS = 2009
REGIMEN = IMIDS_ALKAY

	Probability	CI 95% LOW	CI 95% HIGH
66 - 69	3%	1%	5%
70 - 74	5%	2%	7%
75 - 79	4%	2%	6%
80 - 84	7%	3%	10%
85 +	5%	2%	8%

Appendix Table B. Treatment Choice (Adjusted) by age group

YEAR OF DIAGNOSIS = 2010
REGIMEN = PI_ALKAY

	Probability	CI 95% LOW	CI 95% HIGH
66 - 69	24%	18%	31%
70 - 74	17%	13%	22%
75 - 79	14%	10%	18%
80 - 84	12%	8%	16%
85 +	10%	6%	15%

YEAR OF DIAGNOSIS = 2010
REGIMEN = PI_IMIDS

	Probability	CI 95% LOW	CI 95% HIGH
66 - 69	9%	5%	13%
70 - 74	10%	6%	14%
75 - 79	15%	10%	20%
80 - 84	8%	5%	12%
85 +	9%	4%	14%

YEAR OF DIAGNOSIS = 2010
REGIMEN = PI

	Probability	CI 95% LOW	CI 95% HIGH
66 - 69	20%	14%	25%
70 - 74	21%	16%	26%
75 - 79	21%	16%	26%
80 - 84	24%	18%	30%
85 +	22%	15%	28%

YEAR OF DIAGNOSIS = 2010
REGIMEN = IMID

	Probability	CI 95% LOW	CI 95% HIGH
66 - 69	26%	20%	32%
70 - 74	32%	26%	37%
75 - 79	27%	22%	33%
80 - 84	33%	26%	39%
85 +	32%	25%	40%

YEAR OF DIAGNOSIS = 2010
REGIMEN = IMIDS_ALKAY

	Probability	CI 95% LOW	CI 95% HIGH
66 - 69	4%	1%	6%
70 - 74	6%	3%	8%
75 - 79	5%	2%	7%
80 - 84	9%	5%	12%
85 +	7%	3%	10%

Appendix Table B. Treatment Choice (Adjusted) by age group

YEAR OF DIAGNOSIS = 2011
REGIMEN = PI_ALKAY

	Probability	CI 95% LOW	CI 95% HIGH
66 - 69	32%	25%	40%
70 - 74	24%	18%	30%
75 - 79	19%	14%	24%
80 - 84	17%	11%	22%
85 +	14%	9%	20%

YEAR OF DIAGNOSIS = 2011
REGIMEN = PI_IMIDS

	Probability	CI 95% LOW	CI 95% HIGH
66 - 69	9%	5%	13%
70 - 74	10%	6%	14%
75 - 79	15%	10%	20%
80 - 84	9%	5%	12%
85 +	9%	5%	14%

YEAR OF DIAGNOSIS = 2011
REGIMEN = PI

	Probability	CI 95% LOW	CI 95% HIGH
66 - 69	20%	14%	26%
70 - 74	22%	17%	27%
75 - 79	22%	17%	27%
80 - 84	26%	20%	32%
85 +	23%	16%	29%

YEAR OF DIAGNOSIS = 2011
REGIMEN = IMID

	Probability	CI 95% LOW	CI 95% HIGH
66 - 69	20%	15%	25%
70 - 74	26%	20%	31%
75 - 79	22%	17%	27%
80 - 84	27%	21%	33%
85 +	26%	20%	33%

YEAR OF DIAGNOSIS = 2011
REGIMEN = IMIDS_ALKAY

	Probability	CI 95% LOW	CI 95% HIGH
66 - 69	2%	1%	3%
70 - 74	3%	1%	5%
75 - 79	3%	1%	4%
80 - 84	5%	2%	8%
85 +	4%	1%	6%
