# Supplementary material for: Utilization of novel systemic therapies for multiple myeloma: A retrospective study of front‐line regimens using the SEER‐Medicare data
Source: Cancer Med. 2019 Dec 4;9(2):626–39. doi: 10.1002/cam4.2698 (PMC6970041; doi:10.1002/cam4.2698)
Supplement: Supplementary file 2 [file CAM4-9-626-s002.pdf]

APPENDIX A: ALGORITHM TO IDENTIFY TREATMENT EPISODES

PRESCRIPTION AND ADMINISTRATION EVENTS

We identified prescription and administration events for the time period of 12 months prior to diagnosis date through end of observation. We identified all MEDPAR (Medicare Provider Analysis and Review), NCH (National Claims History), Outpatient, HHA (Home Health Agency), DME (durable medical equipment), PDE (Prescription Drug Event) claims for chemotherapy, radiation, and stem cell transplant using HCPCS (Healthcare Common Procedure Coding System), NDCs (National Drug Codes), and ICD (International Statistical Classification of Diseases and Related Health Problems). (Hospice claims were not used because patients in hospice would not be actively receiving treatment for multiple myeloma.) If an event is missing a start date (Center date or First expense date) in Outpatient, NCH, DME, HHA, Hospice claims, we used "Claim From" Date. If the Last Expense Date was missing, we used the Claim Thru Date. If an event is missing the start date, we used the end date. If an event is missing an end date, we used the start date.

TREATMENT LINE INDENTIFICATION

Anticancer systemic therapy for Multiple Myeloma (MM) includes: bortezomib, carfilzomib, cyclophosphamide, doxorubicin (includes liposomal doxorubicin), interferon, lenalidomide, melphalan, pomalidomide, thalidomide, vincristine, steroids (dexamethasone, and prednisone), and other chemotherapy agents initiated after MM diagnosis

We first bucket continuous drug claims (consisting of one or more drugs listed above) into treatment episode (TXe). Each treatment episode is labeled with (n), where n = the temporal sequence from 1 to x of TXes after MM diagnosis

A treatment gap over 3 months between TXes signals the start of a 2nd line treatment, irrespective of whether the 2nd line treatment is a switch or a retreatment (same regimen as the 1st line treatment))

If a switch of one or more drugs happens after 90 days of the initiation of the initial treatment episode, the switch constitutes a new line of treatment

If a switch of one or more drugs happens before the 90th day of the initiation of a treatment, the initial treatment is a failed treatment and we evaluate the next treatment episode to identify the first line treatment. If the subsequent treatment episode was a failed treatment, we evaluate the following treatment episode. We repeat the process to find the first-line treatment.
