# Supplementary material for: Utilization of novel systemic therapies for multiple myeloma: A retrospective study of front‐line regimens using the SEER‐Medicare data
Source: Cancer Med. 2019 Dec 4;9(2):626–39. doi: 10.1002/cam4.2698 (PMC6970041; doi:10.1002/cam4.2698)

Appendix Figure A: Adjusted Probabilities of Receiving Treatment with confidence intervals (Diagnosis Years = 2008 – 2010)

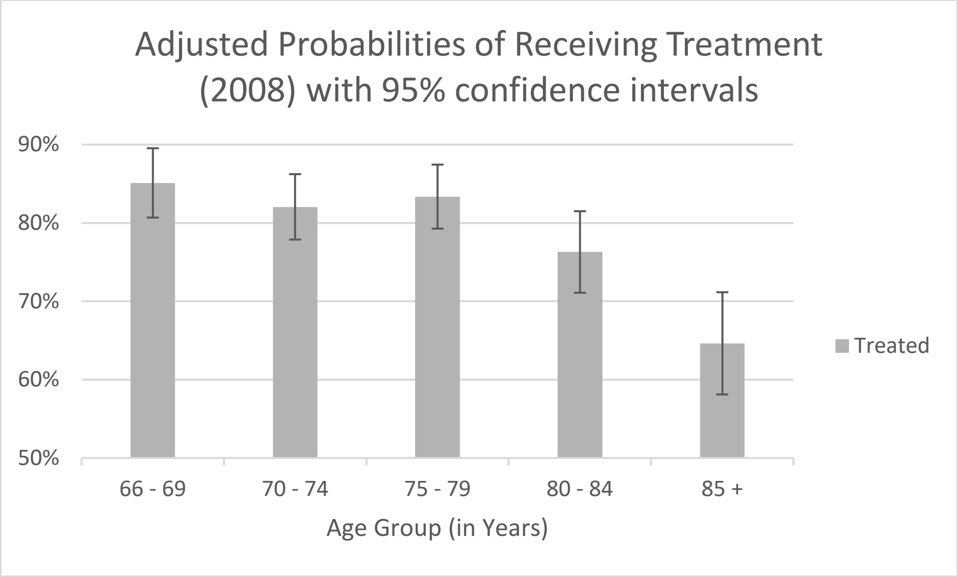

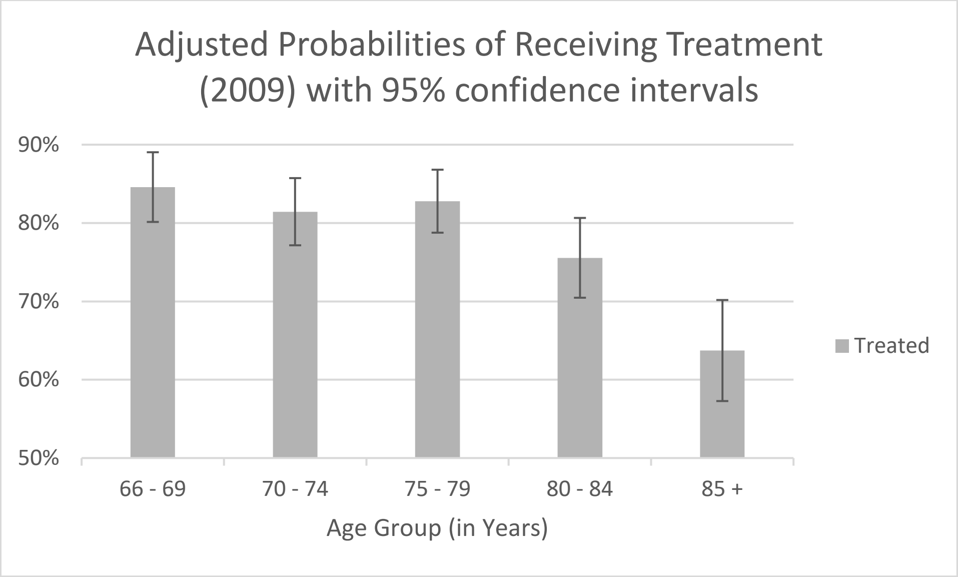

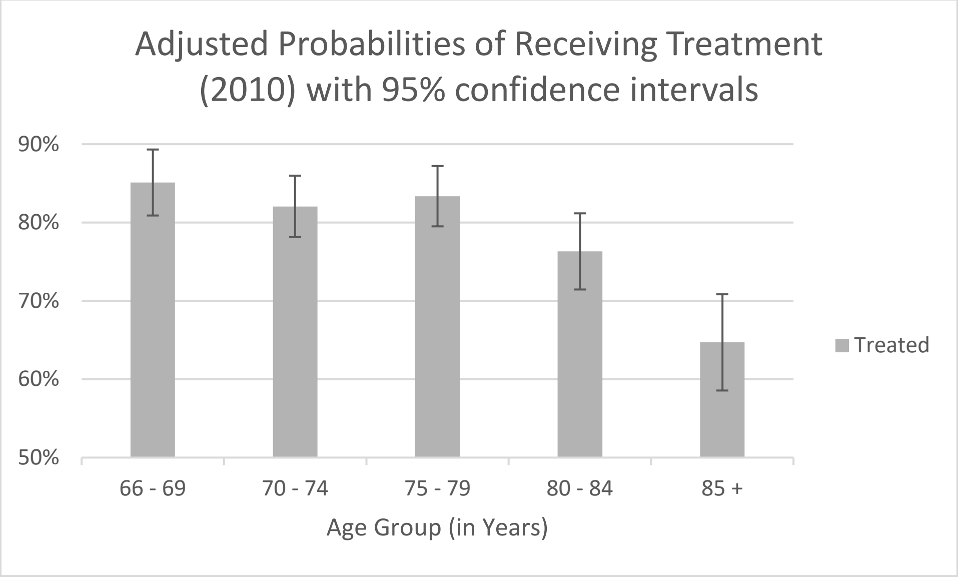

Supplement: Supplementary file 3 [file CAM4-9-626-s003.pdf]
